# Supplementary material for: Low effective mechanical advantage of giraffes’ limbs during walking reveals trade-off between limb length and locomotor performance
Source: Proc Natl Acad Sci U S A. 2022 Jul 7;119(28):e2108471119. doi: 10.1073/pnas.2108471119 (PMC9282232; doi:10.1073/pnas.2108471119)
Supplement: Supplementary File [file pnas.2108471119.sapp.pdf]

PNAS
www.pnas.org

Supplementary Information for

Low effective mechanical advantage of giraffes' limbs during walking reveals trade-off between limb length and locomotor performance

Christopher Basu[1,2] and John R. Hutchinson[2]

[1]Department of Comparative Biomedical Sciences, School of Veterinary Medicine, University of Surrey, Guilford GU2 7XH, UK

[2]Stucture & Motion Laboratory, Department of Comparative Biomedical Sciences, Royal Veterinary College, Hawkshead Lane, North Mymms, Hatfield, Hertfordshire AL9 7TA, UK

Corresponding author Christopher Basu

This PDF file includes:

Supplementary text

Figures S1 to S12

Tables S1 to S4

SI References

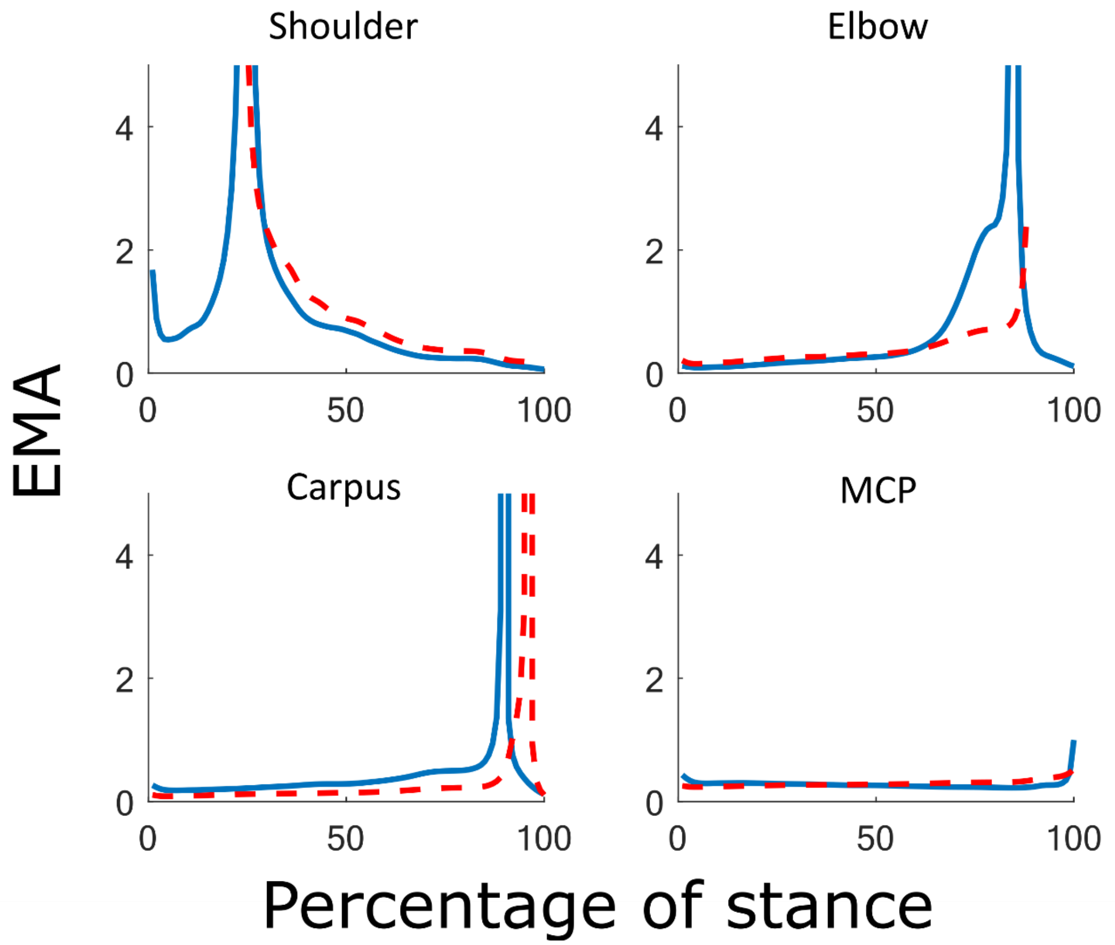

Figure S1 Giraffe forelimb EMA_{imp} derived from experimentally measured kinematics and kinetics (solid blue line) and inverse dynamics, from an exemplar stance phase, compared with EMA_{stat}; estimated from rigid skeletal models (red dotted line). Modelled EMA_{stat} at the start of the shoulder timeseries and end of the elbow timeseries were not modeled, as the muscle moment arms during these times could not reliably be measured from skeletal specimens alone.

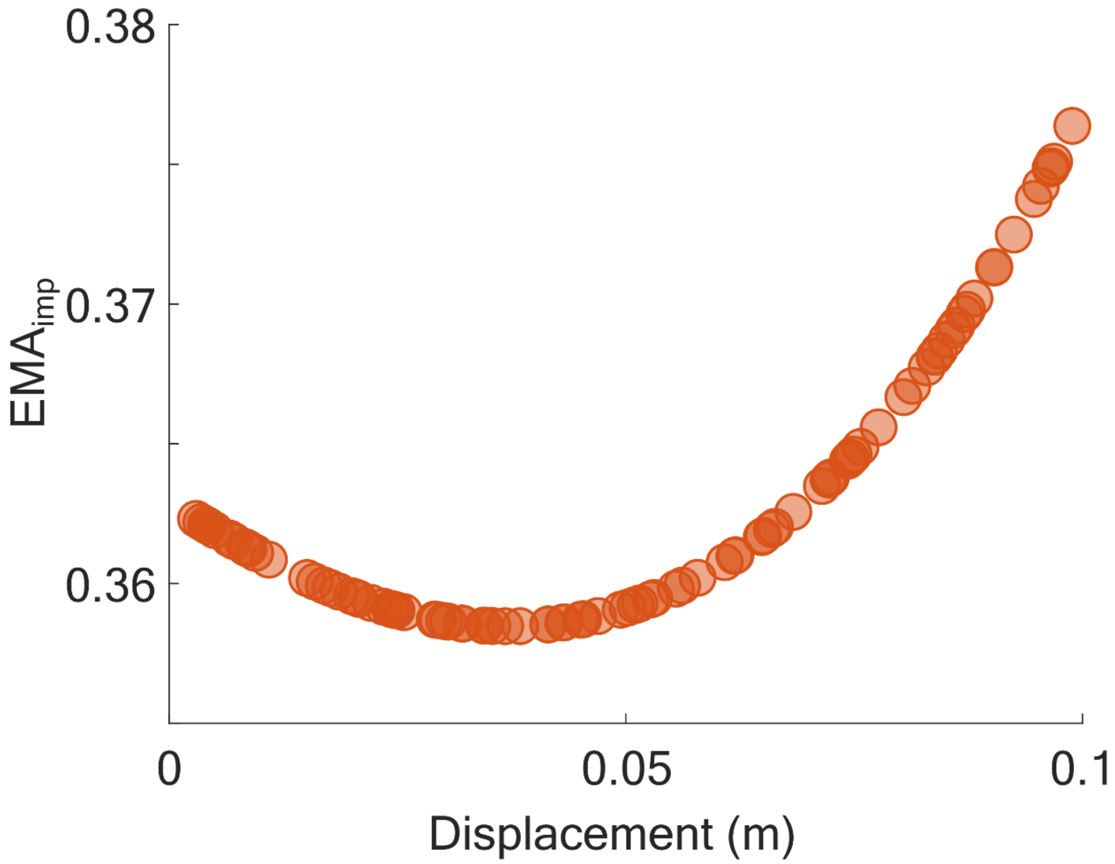

Figure S2 EMA$_{imp}$ varied with displacement of center of pressure location. Regardless of the location under the foot, EMA$_{imp}$ in the giraffe forelimb (0.34 ± 0.05) was four times below predictions for a large cursorial mammal.

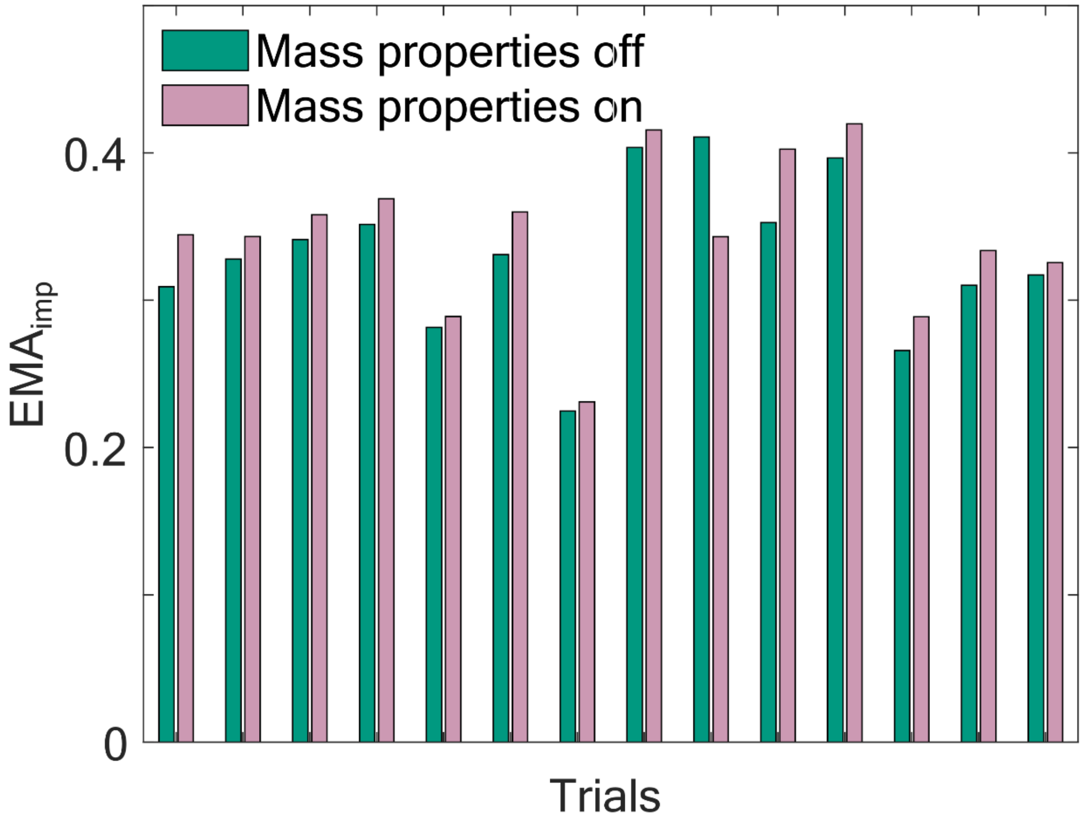

Figure S3 Comparison of EMA$_{imp}$ derived using a massless musculoskeletal model using experimental data from 14 steps ("Trials") of an individual *Giraffa*, with a model featuring mass properties. EMA$_{imp}$ derived using inverse dynamics is comparable regardless of the model used (t-test, p = 0.26).

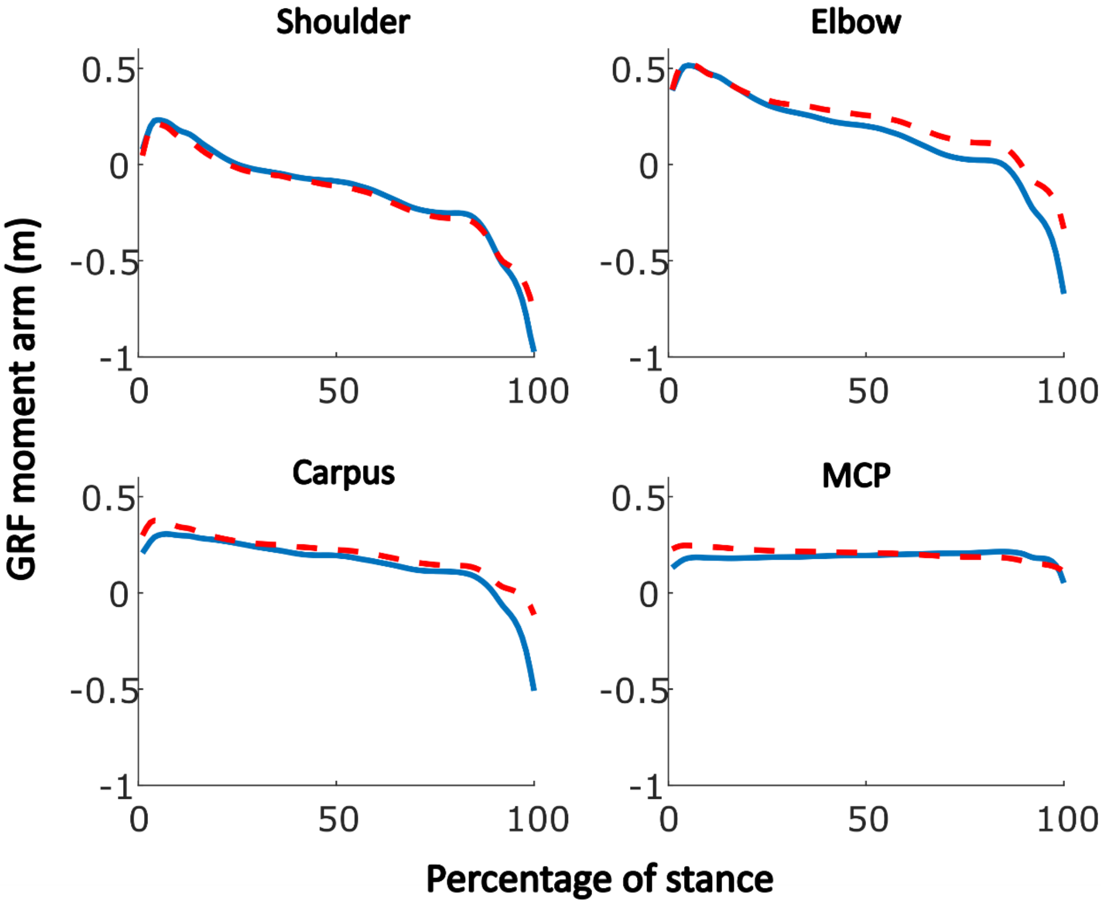

Figure S4 The GRF moment arm (R), with respect to (A) shoulder, (B) elbow, (C) carpus and (D) the MCP joint in the giraffe. The solid blue line shows experimentally derived data from an exemplar trial, compared with the red dashed line which shows the moment arms derived from the static model. The two timeseries show comparable patterns of change, with minor error attributable to positional differences between the joint centres (experimental vs modeled). The GRF moment arms from the two methods, summarized as the mean, show a RMSE of 6%.

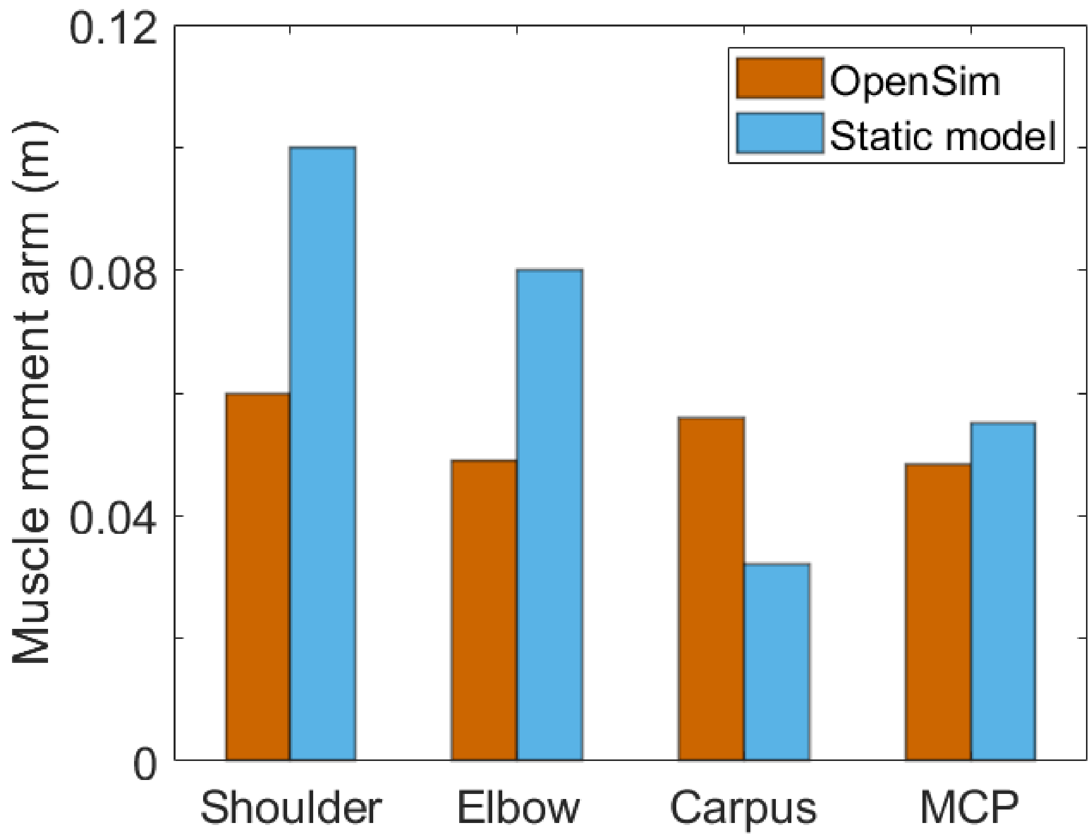

Figure S5 Comparison of muscle moment arms (r) obtained from the giraffe static skeletal model (blue), compared with muscle moment arms exported from the OpenSim musculoskeletal model (orange). Bars refer to shoulder extensors, elbow extensors, carpal flexors, and MCP flexors. Overall, the static modeling approach tended to overestimate moment arms, especially for the shoulder (but not for the carpus).

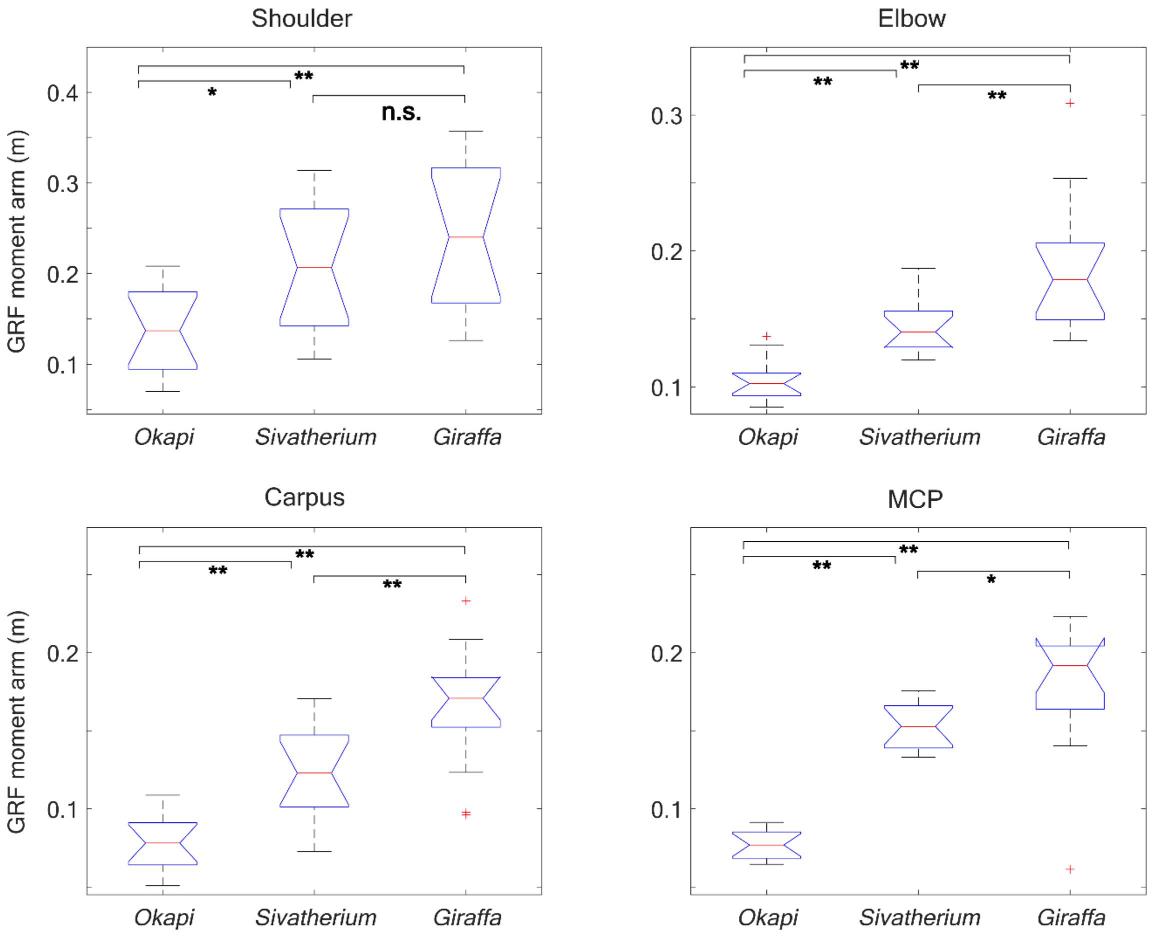

Figure S6 Comparison of absolute GRF moment arms (R) between giraffids, expressed as the time-averaged integral of the moment arms. Differences were evaluated by a one-way ANOVA, and are reported as not significant (n.s.), p<0.05 (*) or p<0.01 (**). These absolute GRF moment arms tended to be larger in larger giraffids.

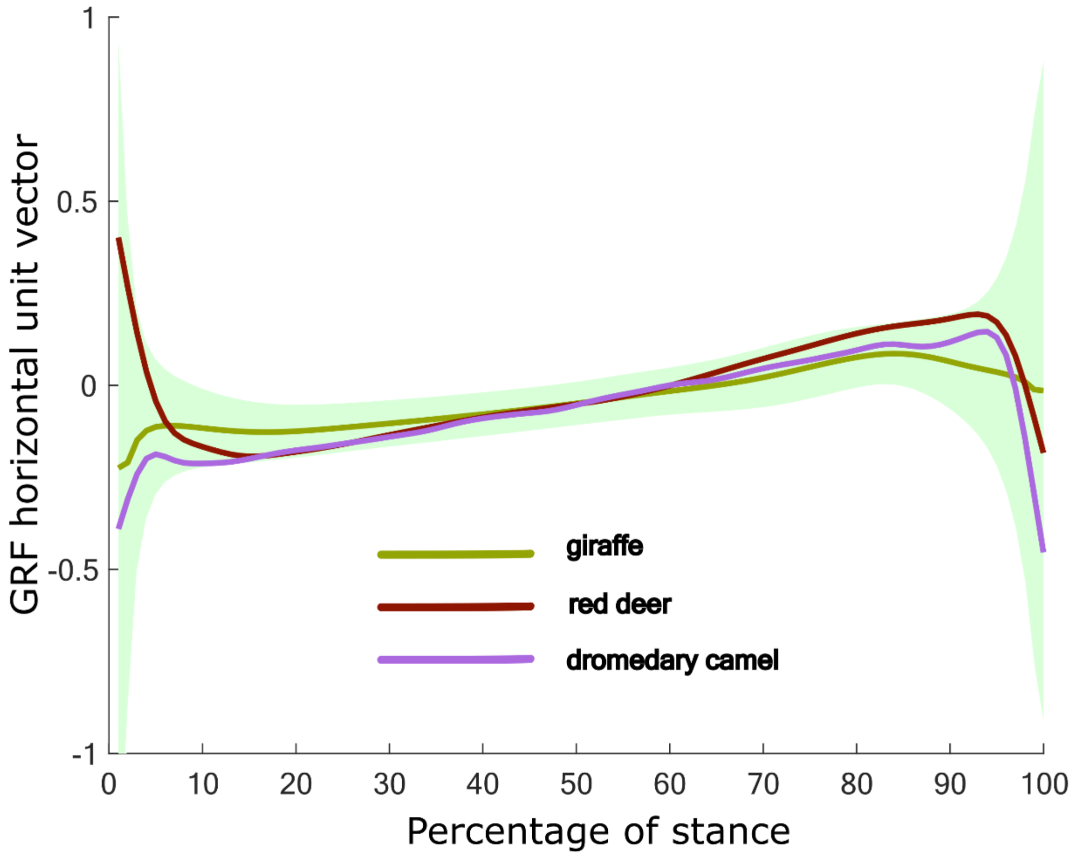

Figure S7 Horizontal GRF unit vectors during the stance phase, in the giraffe (green), red deer (dark red) and dromedary camel (pink). The mean of 46 trials in the case of the giraffe is shown (1), with the shaded region showing the variation (two standard deviations) observed. The data for the deer and dromedary are from one step each (2). The vertical GRF components in each species remained consistently >0.97 when the limb was fully loaded, contrasting with the horizontal GRF component which changed sign from a negative (braking) to positive (acceleratory) vector. The GRF unit vectors for red deer and dromedary fell within the variation seen in giraffes.

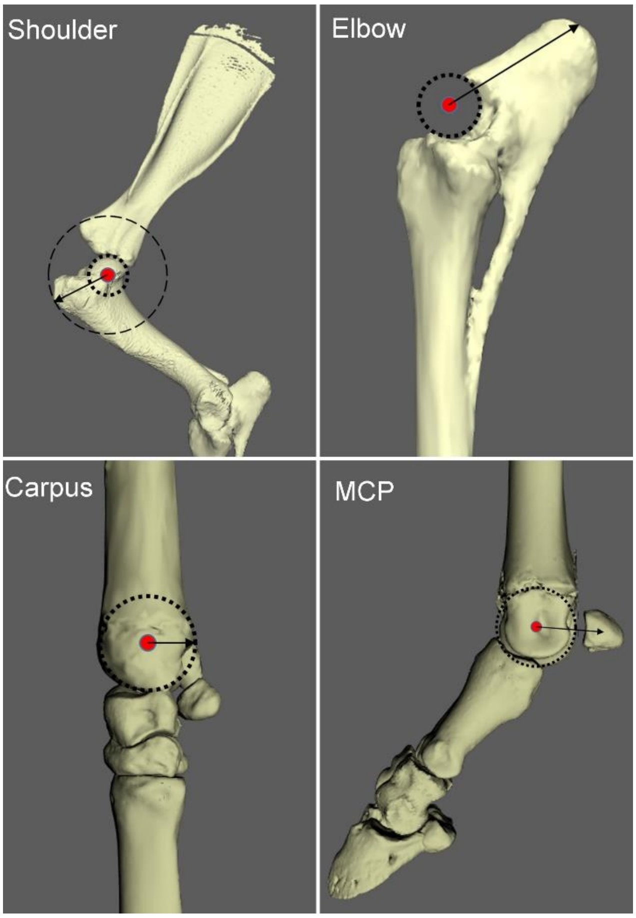

Figure S8 Bone geometry alone was used as a measure of left forelimb (lateral view) muscle moment arms in *Giraffa* (shown), *Sivatherium* and *Okapia*. Dotted lines represent shape fitting to the joint surface, which facilitated measurement of the moment arms (black arrows) in the parasagittal plane. The shoulder extensor moment arm was defined by the distance from the joint center to the greater tubercle. This vector is perpendicular to the path taken by the biceps brachii muscle, as it wraps around the shoulder joint. The orientation of the elbow moment arm was defined using a previous methodology (3). The length of the moment arm was defined by the center point of the corresponding bony region (bounded by the two small red dots). The carpus flexor moment arm was defined by the distance from the center of rotation to the fitted circle. The MCP flexor moment arm was defined as the distance from the center of rotation to the middle of the proximal sesamoid bones.

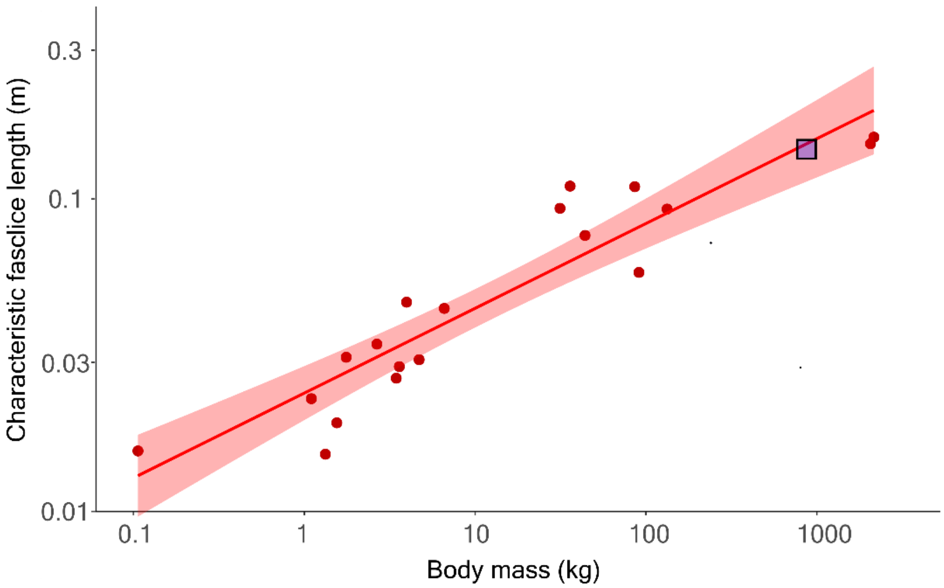

Figure S9 Characteristic muscle fascicle length in terrestrial mammals(4) shows negative allometry. All graphics in red are reproduced from Bishop 2021 (4). Circles are datapoints, with the line showing phylogenetic regression and 95% confidence intervals. Characteristic fascicle length for a 880 kg giraffe cadaver is plotted (square blue datapoint) and falls within the confidence intervals.

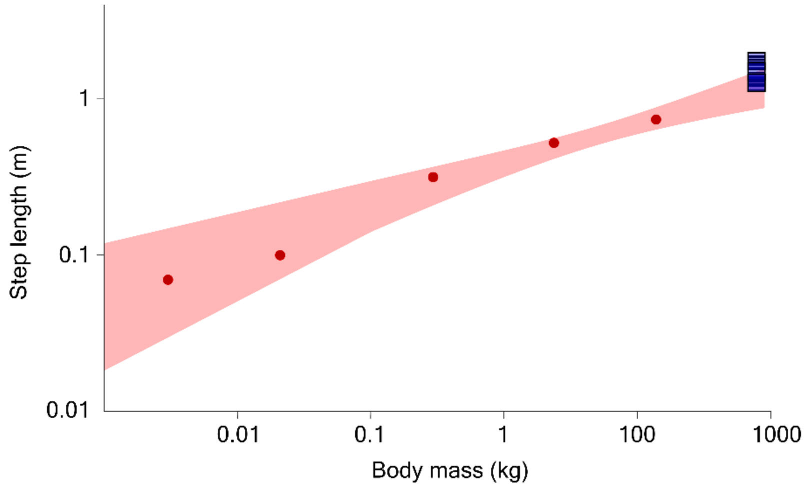

Figure S10 Data showing isometry of step length at moderate running speed (5), with datapoints in red. Data for slow walking giraffes are plotted (blue square markers), and partly fall within the 95% prediction interval (shaded area) of the scaling model (y = 0.10x$^{0.29}$). Longer step lengths were observed at faster walking speeds, as well as at running speeds (6), indicating that giraffes likely take relatively longer steps for their body mass.

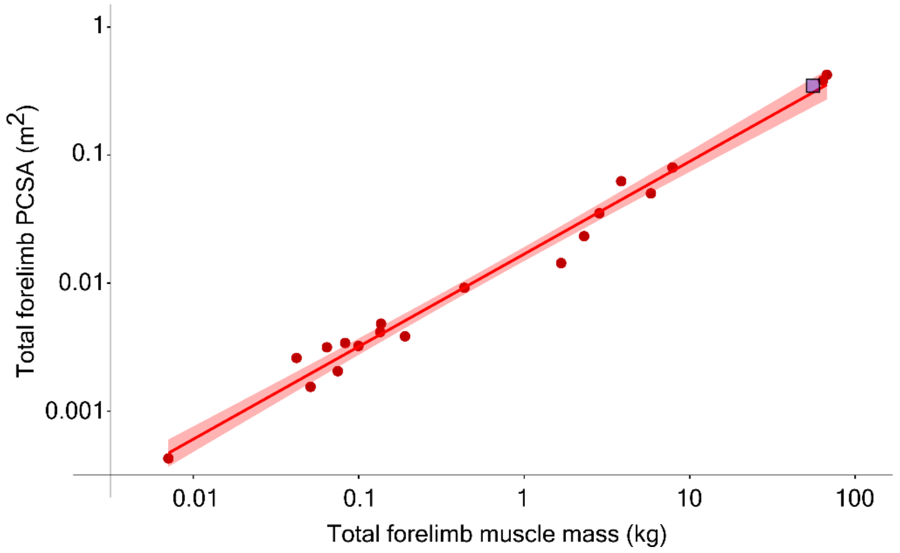

Figure S11 Forelimb muscle PSCA shows positive allometry with respect to muscle mass in terrestrial mammals, although the scaling exponent (0.72) suggests diminished maximal force production in larger mammals. All graphics in red are reproduced from Bishop 2021 (4). Circles are datapoints, with the line showing phylogenetic regression and 95% confidence intervals. Total forelimb PCSA for a 880 kg giraffe is plotted (blue square datapoint) and falls within the confidence intervals, indicating that giraffes follow similar force production constraints as other large mammals.

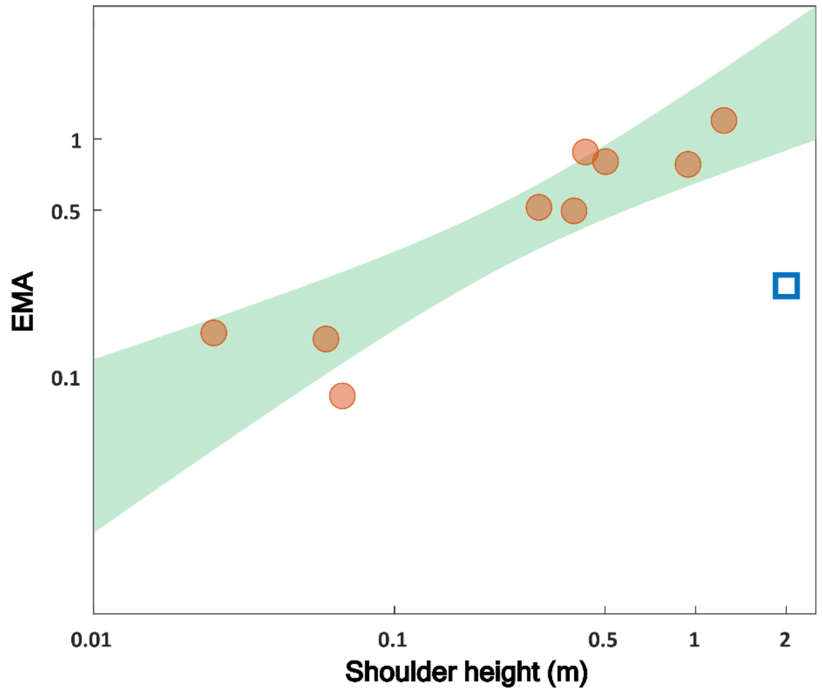

Figure S12 Data from Biewener, showing EMA in a variety of quadrupedal mammals (red points), replotted as a function of limb length , where $y = 1.02x^{0.56}$. Giraffe EMA (blue square) falls below the prediction interval. EMA increases from small mammals to approximate horse size, and is associated with limb segment elongation linked to postural realignment (i.e. limb straightening). Animals in excess of horse size have little opportunity for further limb straightening (hence have constrained EMA increases).

Muscle groups active during stance	Total PSCA (m^2)	Weighted mean fascicle length (m)	Weighted mean muscle moment arm (m)	Static model muscle moment arms (m)		
				Giraffa	*Sivatherium*	*Okapia*
Shoulder extensors	0.10	0.228	0.065	0.10	0.13	0.07
Shoulder flexors	0.076	0.114	0.14	Not measured	Not measured	Not measured
Elbow extensors	0.14	0.116	0.062	0.08	0.15	0.05
Carpal flexors	0.021	0.106	0.060	0.032	0.06	0.02
MCP flexors	0.040	0.059	0.059	0.058	0.036	0.02

Table S1 Muscle architectural data from a 880 kg giraffe. Muscle groups with their total physiological cross-sectional area (PCSA), weighted mean fascicle length l_{fasc}, and mean muscle moment arm (weighted by each muscle's contribution to PCSA) during the stance phase. Comparable measurements from the static skeletal models are shown for comparison.

Specimen number	Description of specimen	Intact/Partial fragment
NHMUK PV OR 39536	Left scaphoid carpal (radial carpal)	Intact
NHMUK PV OR 39537	Left lunar carpal (intermediate carpal)	Intact
NHMUK PV OR 39538	Right cuneiform carpal (ulnar carpal)	Intact
NHMUK PV OR 15695	Left magnum carpal (C2+3)	Intact
NHMUK PV OR 39540	Left unciform carpal (C4)	Intact
NHMUK PV OR 17089	Right MC with articulated magnum	Intact
NHMUK PV OR 39541	Proximal phalanx	Intact
NHMUK PV OR 15805	Second phalanx	Intact
NHMUK PV OR 39534	Right radioulnar	Intact
NHMUK PV OR 39688	Left humerus	Intact
NHMUK PV OR 36680	Left scapula	Partial

Table S2 Specimen details for *Sivatherium giganteum.* All specimens are held at the Natural History Museum (NHMUK), South Kensington, UK.

Joint	Internal angle, flexor aspect (°)
Shoulder	105
Elbow	126
Carpus	181
MCP	198

Table S3 ***Okapia johnstoni*** **midstance angles (7) for walking.**

Actuator	Function		
Anconeus	elbow extensor		
Biceps brachii	shoulder extensor	elbow flexor	
Brachialis	elbow flexor		
Brachiocephalicus	limb protractor	shoulder	
Common digital extensor	elbow flexor	carpus extensor	MCP extensor
Coracobrachialis	shoulder flexor		
DDF	elbow extensor	carpus flexor	MCP flexor
Deep pectineus	limb adductor		
Deltoid	shoulder flexor		
Extensor carpi radialis	elbow flexor	carpus extensor	
Extensor carpi ulnaris	elbow extensor	carpus flexor	
Flexor carpi radialis	elbow extensor	carpus flexor	
Flexor carpi ulnaris	elbow extensor	carpus flexor	
Infraspinatous	shoulder extensor		
Interossous (tendinous derivative)	carpus flexor	MCP flexor	
Lateral digital extensor	elbow extensor	carpus flexor	MCP extensor
Latissimus dorsi	scapula retractor	shoulder flexor	
Oblique carpal extensor	carpus extensor		
Omotransversarius	scapula protractor		
Rhomboid	limb protractor		
SDF	elbow extensor	carpus flexor	MCP flexor
Subscapularis	shoulder extensor		
Superficial pectineus	limb adductor	shoulder flexor	
Supraspinatous	shoulder extensor		
Terres major	shoulder flexor		
Terres minor	shoulder extensor		
Trapezius1	limb protractor		
Trapezius2	limb retractor		
Lateral head of tripeps brachii	elbow extensor		
Long head of triceps brachii	shoulder flexor	elbow extensor	
Medial head of triceps brachii	elbow extensor		

Table S4 31 musculotendon actuators were included in the OpenSim giraffe forelimb musculoskeletal model. Muscle geometry was simplified by grouping multiple heads as one (e.g. lateral and medial heads of the biceps brachii) if they shared similar origin and insertion points. Muscles with broad attachments (e.g. trapezius) where subdivided into discrete units. Each muscles' function was designated on the basis of its action on the forelimb segments.

SI References

1. C. Basu, A. M. Wilson, J. R. Hutchinson, The locomotor kinematics and ground reaction forces of walking giraffes. *Journal of Experimental Biology* **222** (2019).
2. S. E. Warner *et al.*, Size-related changes in foot impact mechanics in hoofed mammals. *PloS one* **8**, e54784 (2013).
3. S.-i. Fujiwara, Olecranon orientation as an indicator of elbow joint angle in the stance phase, and estimation of forelimb posture in extinct quadruped animals. *Journal of morphology* **270**, 1107-1121 (2009).
4. P. J. Bishop, M. A. Wright, S. E. Pierce, Whole-limb scaling of muscle mass and force-generating capacity in amniotes. *PeerJ* **9**, e12574 (2021).
5. R. Kram, C. R. Taylor, Energetics of running: a new perspective. *Nature* **346**, 265-267 (1990).
6. C. K. Basu, F. Deacon, J. R. Hutchinson, A. M. Wilson, The running kinematics of free-roaming giraffes, measured using a low cost unmanned aerial vehicle (UAV). *PeerJ* **7**, e6312 (2019).
7. K. D'Aout, C. Marien, K. Leus, P. Aerts (2005) Gait patterns and hoof impact in a captive giraffid, the Okapi (Okapia johnstoni). in *COMPARATIVE BIOCHEMISTRY AND PHYSIOLOGY A-MOLECULAR & INTEGRATIVE PHYSIOLOGY* (ELSEVIER SCIENCE INC 360 PARK AVE SOUTH, NEW YORK, NY 10010-1710 USA), pp S148-S148.
